# Down-Regulation of 5-HT_1B_ and 5-HT_1D_ Receptors Inhibits Proliferation, Clonogenicity and Invasion of Human Pancreatic Cancer Cells

**DOI:** 10.1371/journal.pone.0105245

**Published:** 2014-08-29

**Authors:** Nilgun Gurbuz, Ahmed A. Ashour, S. Neslihan Alpay, Bulent Ozpolat

**Affiliations:** 1 Department of Experimental Therapeutics, The University of Texas, M.D. Anderson Cancer Center, Houston, Texas, United States of America; 2 Department of Pharmacology and Toxicology, Faculty of Pharmacy, Al-Azhar University, Cairo, Egypt; 3 Non-Coding RNA, The University of Texas, M.D. Anderson Cancer Center, Houston, Texas, United States of America; University of Alabama at Birmingham, United States of America

## Abstract

Pancreatic ductal adenocarcinoma is characterized by extensive local tumor invasion, metastasis and early systemic dissemination. The vast majority of pancreatic cancer (PaCa) patients already have metastatic complications at the time of diagnosis, and the death rate of this lethal type of cancer has increased over the past decades. Thus, efforts at identifying novel molecularly targeted therapies are priorities. Recent studies have suggested that serotonin (5-HT) contributes to the tumor growth in a variety of cancers including prostate, colon, bladder and liver cancer. However, there is lack of evidence about the impact of 5-HT receptors on promoting pancreatic cancer. Having considered the role of 5-HT-1 receptors, especially 5-HT_1B_ and 5-HT_1D_ subtypes in different types of malignancies, the aim of this study was to investigate the role of 5-HT_1B_ and 5-HT_1D_ receptors in PaCa growth and progression and analyze their potential as cytotoxic targets. We found that knockdown of 5-HT_1B_ and 5-HT_1D_ receptors expression, using specific small interfering RNA (siRNA), induced significant inhibition of proliferation and clonogenicity of PaCa cells. Also, it significantly suppressed PaCa cells invasion and reduced the activity of uPAR/MMP-2 signaling and Integrin/Src/Fak-mediated signaling, as integral tumor cell pathways associated with invasion, migration, adhesion, and proliferation. Moreover, targeting 5-HT_1B_ and 5-HT_1D_ receptors down-regulates zinc finger ZEB1 and Snail proteins, the hallmarks transcription factors regulating epithelial-mesenchymal transition (EMT), concomitantly with up-regulating of claudin-1 and E-Cadherin. In conclusion, our data suggests that 5-HT_1B_– and 5-HT_1D_–mediated signaling play an important role in the regulation of the proliferative and invasive phenotype of PaCa. It also highlights the therapeutic potential of targeting of 5-HT_1B/1D_ receptors in the treatment of PaCa, and opens a new avenue for biomarkers identification, and valuable new therapeutic targets for managing pancreatic cancer.

## Introduction

Pancreatic cancer (PaCa), which has a strong invasive capacity with frequent metastasis and recurrence, is known to be one of the most lethal human cancers with <5% 5-year survival rate [1]. Although it only ranks tenth in incidence among the most common human cancers, PaCa is the fourth leading cause of cancer deaths in Western countries and its death rate has not decreased over the past few decades [2], [3]. Overall, PaCa has about 100% mortality because it is generally detected at advance stages since it typically does not cause any symptoms at earlier stages [4]. PaCa is intrinsically resistant to apoptosis and poorly responds to existing therapeutics, including combination chemotherapeutic regimens [5]. To overcome this global health problem, the investigations are focused on the identification of novel molecular targets to develop new treatment strategies.

The mitogenic neurotransmitter, serotonin (5-HT) was previously known to acts as a growth factor [6] for several types of non-tumoral cells (e.g. vascular smooth muscle cells, lung fibroblasts and renal mesangial cells) [7], [8], and tumor cells (e.g. pancreatic carcinoid cells, small cell lung carcinoma cells and colorectal carcinoma) [9], [10], [11]. Recently, 5-HT has emerged as an important regulator of cell proliferation and tumor growth in a variety of cancer types [12], [13], [14], [15]. During tumor progression, tyrosine hydroxylase, the rate-limiting enzyme in the serotonin biosynthesis pathway, is often up-regulated [16]. Importantly, different 5-HT receptors have been identified (5-HT-1–7) based on their structural, functional and pharmacological characteristics [17], [18]. Six of the families of 5-HT receptors are G-protein-coupled, including Gi: 5-HT-1, Gs: 5-HT-4,6,7, and Gq/11: 5-HT-2,5. Only 5-HT-3 is uniquely a ligand-gated cation channel, related to the nicotinic acetylcholine receptor [16]. 5-HT receptors are further divided into different subtypes, e.g. 5-HT-1 family has five subtypes [18], comprising the 5-HT-1A, -1B, -1D, -1E and -1F receptors and couples preferentially to Gi/o to inhibit cAMP formation [19], [20]. In particular, the human 5-HT_1B_ and 5-HT_1D_ receptors are especially similar in sequence despite being encoded by two distinct genes. The precise function of these receptors remains undefined, and progress toward this has been hampered by the lack of selective ligands [21]. It was previously indicated that the 5-HT-1 receptors are extensively expressed in the human breast cancer [22], prostate cancer [23] and bladder cancer cells [18], which could explain the mitogenic effects of the agonists of these receptor in such cancers. Pancreatic cancer research has mostly focused on the study of gene mutations and signal transduction pathways in pancreatic ductal adenocarcinoma (PDAC) cells, whereas the potential role of neurotransmitter receptors in the development and progression of this deadly neoplastic disease has been largely ignored [4]. Given the potential involvement of 5-HT-1 receptors signaling to the proliferation of several types of cancers, with unknown implications of these receptors on PaCa progression, we investigated here the role of 5-HT_1B_ and 5-HT_1D_ receptors in the proliferation and the invasive phenotype of PaCa.

Local invasion can be considered as an initial and essential step in the malignancy of carcinomas, leading to the generation of usually fatal distant metastasis. For the cancer cells to invade distant tissues, they have to penetrate surrounding extracellular matrices. Such cancer cell/ECM interactions are facilitated by the integrin family of cell adhesion molecules including tyrosine-phosphorylated substrates (the tyrosine kinase Src and focal adhesion kinase) [24]. Integrins are trans-membranous α/β heterodimeric receptors that mediate cell-cell interactions and cell attachment to extracellular matrix (ECM) [25], and they serve as receptors for some ECM proteins (e.g., Fibronectin, Vitronectin, Laminin and Collagen) [26]. Altered integrin activity or substrate affinity can contribute to the neoplastic phenotype. Normally, cellular Src is held in an inactive state, but in several cancer types, abnormal events lead to elevated kinase activity of the protein and cause pleiotropic cellular responses inducing transformation and metastasis [27].

As carcinomas progress, the tumors may lose epithelial morphology and acquire mesenchymal characteristics which contribute to metastatic potential. An epithelial to mesenchymal transition (EMT), a critical process similar to the process of embryonic development, is thought to be an important mechanism for promoting cancer invasion and metastasis [28]. In recent years, the ZEB family of zinc finger transcription factors has been documented as essential players of EMT [29]. The epithelial adhesion protein, E-cadherin, is an active suppressor of invasion and growth of many epithelial cancers, and its down-regulation is considered a hallmark of EMT [30], [31]. E-cadherin is a major target gene of the ZEB family transcriptional repressors. EMT-inducing mediators, such as TCF8, trigger epithelial dedifferentiation by impairing the expression/function of E-cadherin [32]. The E-cadherin repressors may regulate developmental transcriptional programs of EMT in tumor cells predisposing them to invasion and metastasis [33]. Thus, mutations in ZEB encoding genes link these factors to malignant tumor progression [29].

The current study focused on investigating the role of 5-HT_1B_ and 5-HT_1D_ receptors on PaCa proliferation and invasion. Our data demonstrates significant reductions in PaCa cells growth, invasion and correlated downstream signaling in response to down-regulation of these serotonin receptors expressions, suggesting the significant involvement of these receptors in promoting pancreatic cancer.

## Materials and Methods

### Cell lines, culture conditions and reagents

The human pancreatic cancer cell lines were obtained from American Type Culture Collection (Manassas, VA). PANC-1 and MIAPaCa-2 cells were cultured in DMEM/F12 supplemented with 10% FBS. All media contain penicillin and streptomycin (100 units/ml). Cells were maintained at 37°C in a humidified atmosphere containing 5% CO_2_/95% air, and were used between passages 4 and 15. Human pancreatic duct epithelial (HPDE) cells were kindly provided by Dr. Kapil Mehta, Department of experimental Therapeutic, M.D. Anderson Cancer Center, as a generous gift. HPDE cells were maintained in keratinocyte serum-free medium (Keratinocyte-SFM, 1X) containing L-glutamine, and supplemented with prequalified human recombinant Epidermal Growth Factor 1–53 (EGF 1–53) and 25 mg/500 ml Bovine Pituitary Extract (BPE) (Invitrogen/Life Technologies, Carlsbad, CA). NF-κB activation inhibitor II, JSH-23 (4-Methyl-N^1^-(3-phenylpropyl)benzene-1,2-diamine) (EMD Millipore Billerica, MA), was dissolved in DMSO at a final stock concentration of 10 mM, and directly added to cell cultures at 25 and 50 µM concentrations, which selectively blocks nuclear translocation of NF-κB p-65 and its transcription activity.

### Transfections with siRNA

Exponentially growing untreated PANC-1 and MIAPaCa-2 cells were plated 24 h before transfection. Plated cells were transfected with double-stranded siRNA targeting the mRNA of the serotonergic receptors (5-HT-1) subtype –B or –D (Sigma-Aldrich, St. Louis, MO), or transfected with control (non-silencing) siRNA; (5′-AAUUCUCCGAACGUGUCACGU-3′) [34], [35] (Sigma-Aldrich, St. Louis, MO). siRNA targeting tissue transglutaminase (TG2) (Qiagen, Valencia, CA) were also employed [35]. Cells were transfected with either siRNA, at a final concentration of 25–50 nM for 72 h, using HiPerFect Transfection Reagent (Qiagen, Valencia, CA) according to the manufacturer's protocol. The concentrations of siRNAs were chosen based on dose-response studies. Non-silencing control siRNA–transfected cells were used as negative controls. After treatment, the cells were harvested/processed for further analysis and assays.

### Cell viability and proliferation/growth assays

The viability and/or proliferation of cells were detected by MTS assay (Promega, Madison, WI, USA), after cells treatment, to measure cell growth. Cells were counted using a hemocytometer and viable cells were identified by trypan blue exclusion. Viable cells were seeded in 96-well plates (1.5×10^3^ cells/well), and transfected with indicated siRNAs. After 72 h of treatment, a solution containing MTS (3-(4,5-dimethylthiazol-2-yl)-5-(3-carboxy-methoxyphenyl)-2-(4-sulfophenyl)-2H-tetrazolium) and PMS (phenazine methosulfate) (20∶1 v/v) was added to the cells. After 2-3 h of incubation at 37°C, the viable growing cells were estimated by monitoring the absorption of the product at 490 nm, based on the generation of formazan via live cells. All experiments were performed in triplicate and the results were reported as mean of absorption ± standard deviation.

### Clonogenic survival assay

PaCa cells were seeded in 6-wells plates (1.5×10^3^ cells/well), transfected with non-silencing control siRNA, or siRNA against 5-HT_1B_ or 5-HT_1D_ (once/week), and grown for 2 weeks. The formed-colonies were stained with crystal violet and the colonies-area distribution regions were measured densitometrically [36]. Each experiment was performed in triplicate and the results were reported as mean of absorption ± standard deviation.

### Matrigel invasion assay

PaCa cells were transfected with 50 nM of indicated siRNAs, and 72 h later, equal number of the treated viable cells (4×10^4^ cells), were seeded onto Matrigel-coated Transwells (with 8- µm pore size filters) in Matrigel invasion chambers (BD Biosciences, San Jose, CA). The number of cells that invaded the lower side of the membrane after 24 h was determined by counting cells in a minimum of four randomly selected areas. The experiments were performed in triplicate and the results were reported as mean of percentages of invasion ± standard deviation.

### Migration Assay

*In-vitro* wound-healing assay was used to assess cell motility and the ability to migrate. PANC-1 cells were plated in 6-well plates (5×10^5^ cells/well), and cultured in medium containing 10% FBS to achieve a nearly confluent cell monolayer. A scratch was then carefully made on the cell layer using a 10 µL sterile micropipette tip, and any cellular debris was removed by washing with PBS to remove floating cells. The wounded monolayers were then transfected with 50 nM of indicated siRNAs. Immediately after the treatments, the cells were photographed using a phase-contrast microscope (Nikon), to determine the wound width at time 0. The cultures were continued, and the cells were photographed again after 12 h and after 24 h of wounding the cell layer. The Wound healing was visualized by comparing photographs taken at 0 h with those taken at 12 h and 24 h later, and analyzed for the distance migrated by the leading edge of the wound at each time point. The distance traveled by the cells was determined by measuring the wound width at time 12 h and 24 h, and subtracting it from the wound width at time 0. The values obtained were then expressed as % migration, setting the gap width at 0 h as 100%. Three experiments were done in triplicate.

### Western blot analysis

Cells were seeded in 25-cm^2^ culture flasks (0.5×10^6^ cells/flask). Following treatments, the cells were collected, centrifuged, washed twice in ice cold PBS and whole-cell lysates were obtained by suspending the cells in a lysis buffer at 4°C. Lysates were centrifuged at 13,000 g for 10 min at 4°C, and the supernatant fractions were collected. Total protein concentration for each sample was determined by a detergent compatible protein assay kit (Bio-Rad, Hercules, CA), and Western blotting was performed as 40 µg protein/lane on 4–15% SDS-PAGE gel. The proteins were electro-transferred to PVDF membranes and were first incubated with the following primary antibodies; p-Src (Tyr-416), Src, β1 integrin, uPAR, MMP-2, Snail, TCF8/ZEB1, NF-κB (p-105/p-50), and Claudin-1 (Cell Signaling Technology, Danvers, MA); p-FAK (Tyr-397), FAK (BD Biosciences, Franklin Lakes, NJ); 5-HT_1B_ (Sigma Chemical, St. Louis, MO); TG2 and α-SMA (Abcam; Cambridge, MA); Fibronectin and 5-HT_1D_ (Santa Cruz Biotechnology, Santa Cruz, CA), and then with horseradish peroxidase–conjugated anti-rabbit or anti-mouse secondary antibody (Cell Signaling Technology, Danvers, MA). β-actin (Sigma Chemical, St. Louis, MO), or α-β-Tubulin (Cell Signaling Technology, Danvers, MA) were used as loading controls. All antibodies were diluted in TBS-Tween 20 containing 5% dry milk. Chemiluminescent detection was performed with Chemi-glow detection reagents (Alpha Innotech, San Leandro, CA), and the blots were visualized with a FluorChem 8900 imager, and quantified by a densitometer using the image analysis program (ImageJ 1.48s processing software, National Institutes of Health, Bethesda, MD, USA). All experiments were independently repeated at least twice.

### Reverse phase protein arrays (RPPA)

The siRNA-transfected PANC-1 cells (0.5×10^6^ cells/2 ml media) were seeded in 6-well plate. After 72 h incubation, the cells washed twice with PBS, and 150 µL of the lysis buffer [1% Triton X-100, 50 mM Hepes (pH 7.4), 150 mM NaCl, 1.5 mM MgCl_2_, 1 mM EGTA, 100 mM NaF, 10 mM Sod. pyrophosphate, 1 mM Na_3_VO_4_ and 10% glycerol, containing proteinase and phosphatase inhibitors (Roche Applied Science, Indianapolis)] were added to each well. The cell lysates were collected, and RPPA was processed as described before [35].

### RNA isolation and reverse transcriptase–polymerase chain reaction (RT-PCR) analysis

Total RNA was isolated from the collected cells with TRIzol Reagent (Invitrogen/Life Technologies, Carlsbad, CA), and cDNA was obtained from 1 µg of total RNA using RevertAid First Strand cDNA Synthesis Kit (Thermo Scientific). The cDNA for 5-HT_1B_, 5-HT_1D_, β1 integrin, TG2 and GAPDH were amplified using Platinum Taq DNA Polymerase kit (Invitrogen/Life Technologies), with specific primers. Briefly, 2 µL of the total 20 µL of reverse-transcribed product were used for PCR in 1× PCR buffer containing 1.5 mM MgCl_2_, 200 µM deoxynucleotide triphosphates (dNTPs), 1 unit of Platinum Taq polymerase, and 0.2 µM of each indicated primers (Integrated DNA Technologies, IDT), or GAPDH–specific primers (Thermo Scientific). The sequences of the sense and anti-sense 5-HT_1B_ primers are 5′-TGCTGGTTATGCTATTGGCG-3′; and 5′-GATGACACAGAGGTGCAGGATG-3′, respectively. The sequences of the sense and anti-sense 5-HT_1D_ primers are 5′-TGCCGTGGTCCTTTCCGTC-3′; and 5′-GGTGATGGTATAGGCGATGCTG-3′, respectively. The sequences of the sense and anti-sense β1 integrin primers are 5′-CCTACTTCTGCACGATGTGATG-3′; and 5′-CCTTTGCTACGGTTGGTTACATT-3′, respectively. The sequences of the sense and anti-sense TG2 primers are 5′-TAAGAGATGCTGTGGAGGAG-3′; and 5′-CGAGCCCTGGTAGATAAA-3′, respectively. The cDNA samples were incubated at 94°C (2-5 min.) to denature the template and activate the enzyme. This step was followed by 35 cycles of PCR amplification (as 94°C for 30 s, 55°C for 30 s and 72°C for 60 s with 5-HT_1B_ primer; 94°C for 30 s, 60°C for 30 s and 72°C for 60 s with TG2 primer; 94°C for 30 s, 58°C for 45 s and 72°C for 60 s with 5-HT_1D_ and β1 integrin primers, in each cycle). The PCR reaction was terminated with a final extension step of 5 min. at 72°C. The amplified reaction products were analyzed on a 1.2% agarose gel containing ethidium bromide. The cDNA synthesis was verified by detection of the GAPDH transcript, which was used as an internal control.

### Statistical analysis

The data were expressed as the mean ± SD of three independent experiments, and statistical analysis was performed using the Student's *t*-test, to determine statistical significance. P values less than 0.05 were considered statistically significant and are indicated by an asterisk.

## Results

### 5-HT_1B_ and 5-HT_1D_ receptors are overexpressed in pancreatic cancer cells

**Figure 1:**
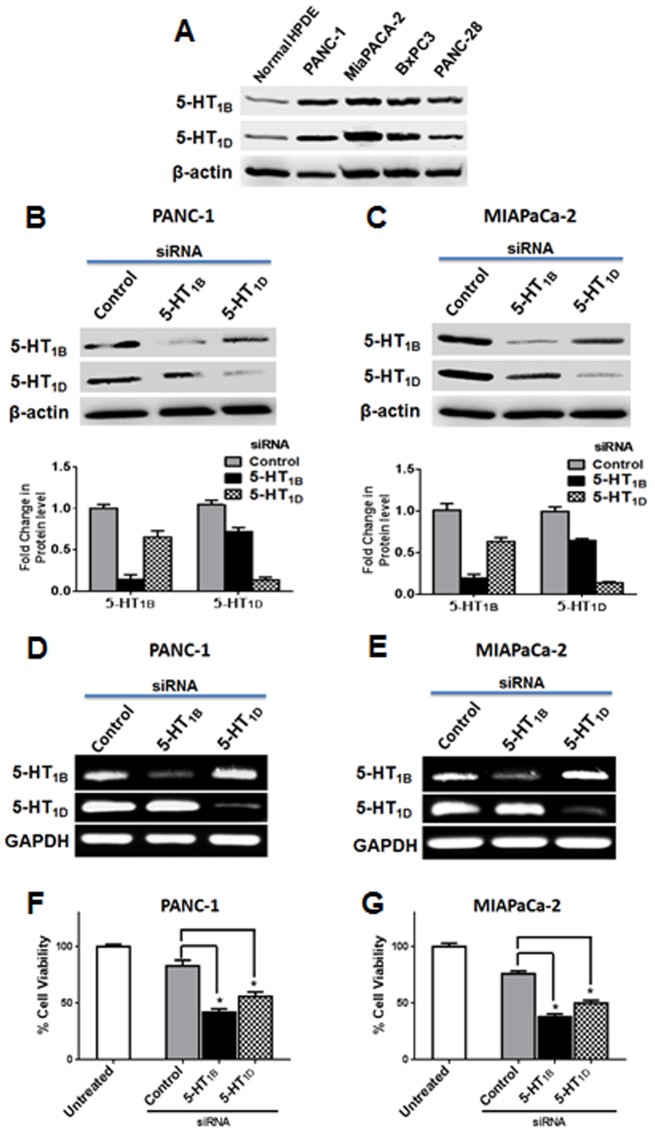
Effects of down-regulation of 5-HT_1B_ and 5-HT_1D_ receptors on PaCa cell proliferation. (**A**) 5-HT_1B_ and 5-HT_1D_ receptors are highly expressed in several pancreatic cancer cell lines. Cell lysates of several PaCa cell lines and normal human pancreatic duct epithelial (HPDE) cells were subjected to Western blot analysis as indicated. β-actin was used as a loading control. (**B–C**) 5-HT_1B_ and 5-HT_1D_ receptors expression levels in PANC-1 (B) and MIAPaCa-2 cells (C) after knockdown of the receptors expression with their corresponding siRNAs. Cells were transfected with 50 nM of indicated siRNAs, and 72 h later, cell lysates were subjected to Western blot analysis. β-actin was used as loading control. The histograms show the relative quantification of the indicated proteins levels. (**D–E**) mRNA expression of 5-HT_1B_ and 5-HT_1D_ receptors in PANC-1 (D) and MIAPaCa-2 cells (E) after knockdown of the receptors expression with their corresponding siRNAs. Cells were transfected with 50 nM of indicated siRNAs, and 72 h later, the total RNA was extracted and the transcript levels of 5-HT_1B_ and 5-HT_1D_ were determined by standard RT-PCR as described in Materials and Methods. GAPDH was used as loading control. (**F–G**) siRNA-mediated 5-HT_1B_ and 5-HT_1D_ receptors knockdown inhibits PaCa cells proliferation. PANC-1 (F) and MIAPaCa-2 cells (G) were transfected with 50 nM of indicated siRNAs, and after 72 h, proliferation was detected by an MTS assay. Data are represented as mean ± SD. * P<0.05 vs. control cells. All experiments were independently performed three times.

Increased 5-hydroxytryptamine biosynthetic capacity as well as severe alterations in the expression patterns of the 5-HT receptors, has been previously reported during progression of breast cancer [16]. Here, we evaluated the expression of 5-HT_1B_ and 5-HT_1D_ receptors in different PaCa cells as well as in normal human pancreatic duct epithelial (HPDE) cells. We found that these receptors are up-regulated in all PaCa cells tested, comparing with its low expression in normal pancreatic epithelium (Fig. 1A), suggesting that the dys-regulation of these receptors might promote signaling favor tumor progression in PaCa cells.

### Knockdown of 5-HT_1B_ and 5-HT_1D_ receptors expression inhibits proliferation/viability of PaCa cells

We aimed to investigate the involvement of these receptors in the proliferation and growth of PaCa cells. Toward this end, we knocked down the expression of each receptor subtype in PANC-1 and MIAPaCa-2 cells, using specific small interfering RNA (siRNA). Despite being encoded by two distinct genes, the human 5-HT_1B_ and 5-HT_1D_ receptors are especially similar in sequence [21]. Therefore, as shown in Figure 1B and C, 5-HT_1B_ siRNA treatments led to a relatively lower expression of 5-HT_1D_ protein level, with a similar lower 5-HT_1B_ protein levels induced by 5-HT_1D_ siRNA. This could be attributed to the fact that both 5-HT_1B_ and 5-HT_1D_ receptors subtypes share a high amino acid sequence identity (∼68% amino acid sequence homology), have similar ligand binding properties and are almost indistinguishable pharmacologically [37]. Such similarities-induced interactions between the two receptor types at the protein level could be occurred after gene expression (e.g, during protein maturation or folding). Therefore, the detecting of the receptors protein expression by Western blotting was not completely sufficient in distinguishing these closely-related receptor subtypes to establish their respective physiological relevance. To overcome such problem in order to investigate the biological effects of targeting each subtype individually, we used RT-PCR analysis and examined the effect of siRNA treatments on the transcription levels of the corresponding gene subtype. Our results clearly show that 5-HT_1B_ specific siRNA and 5-HT_1D_ specific siRNA were confirmed to inhibit the expression of the corresponding mRNA without any significant effect on the other cross analogue subtype in both PANC-1 and MIAPaCa-2 cell lines (Fig. 1D and E). We next analyzed the proliferation after 72 h of siRNA treatment by MTS assay. As shown in Figure 1F and G, our results demonstrated that knockdown of 5-HT_1B_ and 5-HT_1D_ expression significantly inhibited the proliferation of both PANC-1 and MIAPaCa-2 cells. The combined down-regulation of both 5-HT_1B_ and 5-HT_1D_ subtypes impairs proliferation more than down-regulation of either receptor alone (Figure S1), suggesting the biological benefits provided from simultaneous targeting both receptors.

### Targeting 5-HT_1B_ and 5-HT_1D_ receptors inhibits cell clonogenicity of PaCa cells

**Figure 2:**
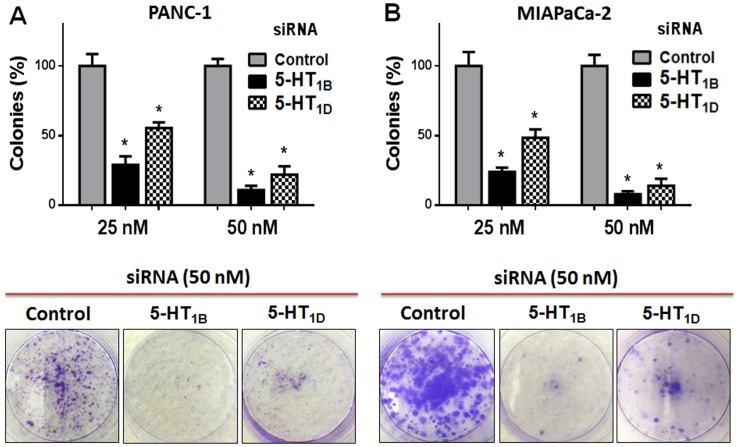
Effect of targeting 5-HT_1B_ and 5-HT_1D_ receptors on PaCa cell clonogenicity. PANC-1 (**A**) and MIAPaCa-2 cells (**B**) were transfected (once/week) with indicated siRNAs. The cells were incubated for 14 days, the colonies were stained with crystal violet and the colonies-area distribution regions were measured densitometrically at the end of the 14 days. The histograms show the percentages of the formed colonies, after 14 days of the first transfection. Data is expressed as mean of percentages of colony formation ± SD of three independent experiments. * P<0.05 vs. control cells.

To further verify the role of 5-HT-1 serotonergic-receptors in PaCa cell proliferation and colony formation, we evaluated the clonogenic capacity of PaCa cells following knock-down of 5-HT_1B_ and 5-HT_1D_ receptors expression. This assay is an *in-vitro* cell survival assay based on the ability of a single cell to grow and form foci into a colony [36]. Knockdown of 5-HT_1B_ and 5-HT_1D_ receptors, using their specific siRNAs, markedly inhibits the ability of PANC-1 and MIAPaCa-2 cells to form colonies (Fig. 2A and B, respectively). Overall, these findings suggest that 5-HT_1B_– and 5-HT_1D_–mediated signaling is involved in the proliferation and clonogenic capability of PaCa cells.

### Targeting 5-HT_1B_ and 5-HT_1D_ receptors impairs cell invasion/migration of PaCa cells

**Figure 3:**
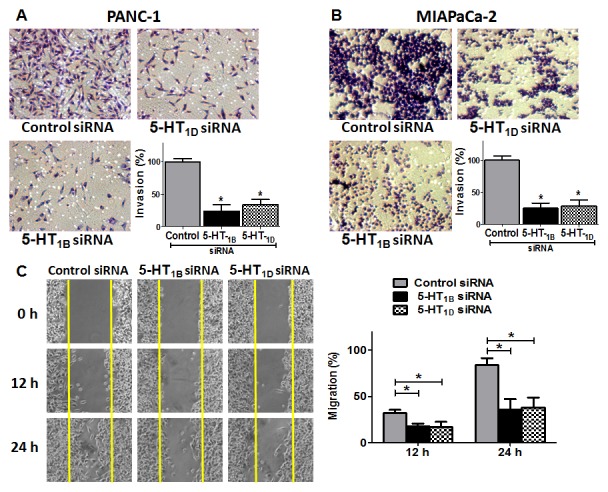
Effect of down-regulation of 5-HT_1B_ and 5-HT_1D_ receptors expression on the invasion/migration of PaCa cells. PANC-1 cells (**A**) and MIAPaCa-2 cells (**B**) were transfected with 50 nM of indicated siRNAs (for 72 h), and equal numbers of viable cells were seeded onto Matrigel-coated Transwell filters in Matrigel invasion chambers. The number of the cells that invaded after 24 h was determined as in protocol. Magnification,100×. The histograms show the mean of percentages of invasion ± SD of three experiments. * P<0.05 vs. control cells. (**C**) The involvement of 5-HT_1B_ and 5-HT_1D_ receptors in regulation of PANC-1 cell motility as analyzed by the wound healing assay. A single scratch was made in the center of the confluent cell monolayer, and the wounded monolayers were transfected with indicated siRNAs. The wounds repair was monitored for 24 h and visualized microscopically with original magnification ×100. Images were taken immediately (0 h), and after 12 h and 24 h of scratching the cultures. The histogram shows the percentages of the cells migration, and the data is expressed as mean of the percentages of migration ± SD of three independent experiments. * represents significant difference between indicated groups (P<0.05).

Pancreatic ductal adenocarcinoma is characterized by highly invasive phenotype and strong metastatic capacity [38]. Because the expression of 5-HT_1B_ and 5-HT_1D_ receptors is elevated in PaCa cells, we assessed whether these receptors are involved in promoting such invasive phenotype. Therefore, we knocked down these receptors in PANC-1 and MIAPaCa-2 cells by siRNAs and evaluated the changes in their invasive capability by *in-vitro* invasion assay using Matrigel-coated Boyden chambers. This assay mimics the *in-vivo* invasion process and measures the number of cancer cells passing through a basement membrane matrix towards media containing chemo-attractants [39]. The most striking finding was that knockdown of 5-HT_1B_ and 5-HT_1D_ receptors significantly reduced the invasion of PANC-1 cells by about 76% and 66%, respectively (Fig. 3A), and reduced the invasion of MIAPaCa-2 cells by about 75% and 71%, respectively (Fig. 3B). We next examined the involvement of 5-HT_1B_ and 5-HT_1D_ receptors in mediating PANC-1 cell motility using the scratch assay at 12 h and 24 h time points. The analysis revealed that the distance covered by migrating cells was significantly decreased when the cells transfected with 5-HT_1B_ or 5-HT_1D_ receptors siRNAs compared to cells exposed to the non-silencing control siRNA (Fig. 3C). These results demonstrate a correlation between PaCa cell motile behavior and 5-HT_1B/1D_ expression. Overall, this data indicates that 5-HT_1B_ and 5-HT_1D_ receptors play a role in mediating PaCa cells migration and invasion.

### 5-HT_1B_ and 5-HT_1D_ receptors are involved in regulation of β1 integrin expression in PaCa Cells

**Figure 4:**
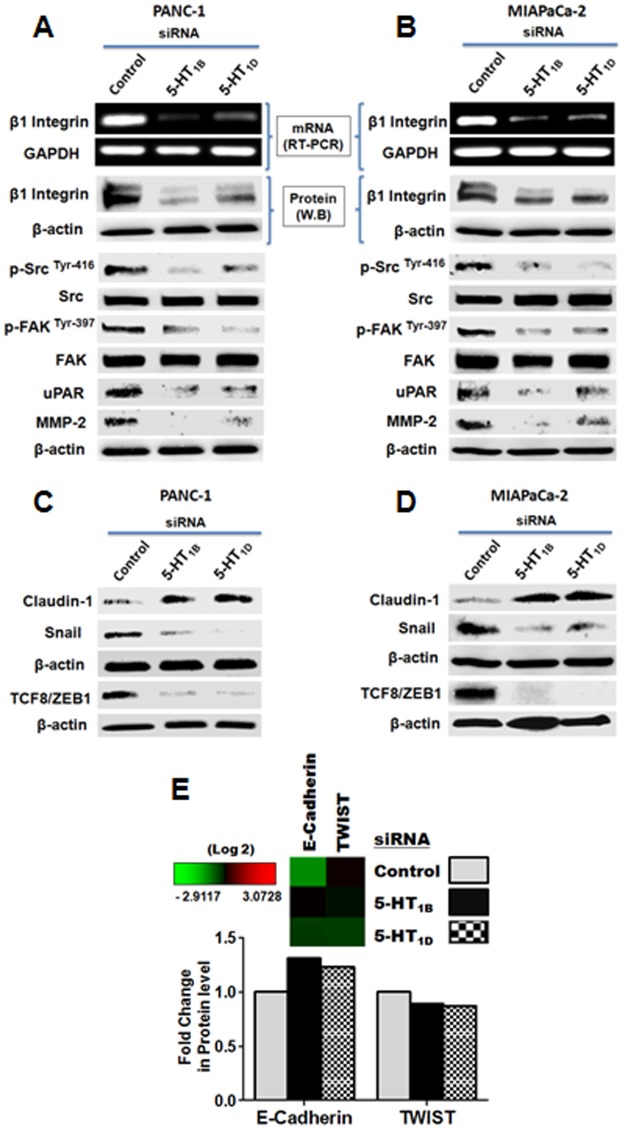
Downstream molecular effects of knockdown of the expression of 5-HT_1B_ and 5-HT_1D_ receptors, on invasion and proliferation biomarkers in PaCa cells. (**A–B**) The effect of siRNAs-mediated 5-HT_1B_ and 5-HT_1D_ receptors down-regulation on β1 integrin/ECM-mediated downstream signaling in PANC-1 (A) and MIAPaCa-2 cells (B). (Upper panel) Cells were transfected with 50 nM of indicated siRNAs, and after 72 h, the total RNA was extracted and the transcript levels of 5-HT_1B_ and 5-HT_1D_ were determined by standard RT-PCR as described in Materials and Methods. GAPDH was used as loading control. (Lower panel) Cells were transfected with 50 nM of indicated siRNAs, and after 72 h, cell lysates were subjected to Western blot analysis. β-actin was used as loading control. (**C–D**) The effect of 5-HT_1B_ and 5-HT_1D_ receptors down-regulation on EMT process in PANC-1 (C) and MIAPaCa-2 cells (D). Silencing of 5-HT_1B_ and 5-HT_1D_ receptors significantly increase the expression of EMT tight junction protein, claudin-1, and decrease the zinc finger ZEB1 transcriptional factors, TCF8 and Snail. The cells treated as in (A–B). Three independent experiments were performed with similar results, and representative data is shown. (**E**) Heat map clustered from RPPA analysis, and a histogram of the normalized fold changes of the selected EMT-markers in PANC-1 cells treated with 50 nM of indicated siRNAs for 72 h. Knockdown of 5-HT_1B_ and 5-HT_1D_ receptors increase the epithelial marker, E-cadherin, and decrease its transcriptional repressor (TWIST).

After finding that 5-HT_1B_ and 5-HT_1D_ receptors are over-expressed in PaCa cells, suggestive of significant alterations in growth-promoting downstream signaling, we next investigated some downstream molecular effects of knockdown of these receptors. The integrin family of trans-membrane receptors links the extracellular matrix (ECM) to the intracellular actin cytoskeleton at focal adhesions interaction points. In addition to this structural role, integrin clustering can initiate intracellular signaling events that promote cell proliferation, survival and migration in both normal and tumorigenic cell contexts [40]. From integrin family, β1-subtype is known to induce Src and FAK activity through the recruitment and activation of Src/FAK dual kinase complex [41]. Because, down-regulation of 5-HT_1B/1D_ receptors suppresses PaCa cells migration/invasion (Fig. 3), we examined whether these receptors regulate the expression of β1 integrin. We employed Western blot and RT-PCR analysis to determine the expression of β1 integrin protein and mRNA levels, respectively, after silencing these receptors. We found that 5-HT_1B_ and 5-HT_1D_ receptors knockdown significantly induce down-regulation of β1 integrin expression at both protein and mRNA level in both PANC-1 and MIAPaCa-2 cells (Fig. 4A and B).

### 5-HT_1B_ and 5-HT_1D_ receptors promote activation of Src–FAK signaling

The tyrosine-phosphorylated substrates (e.g, the non-receptor protein tyrosine kinase Src and focal adhesion kinase) function in integrin signaling [24]. FAK is associated with Src, and they have been shown competent in recruiting/engaging as a major component of an integrin signaling pathway [24], [27]. Integrin-mediated activation of Src/FAK linkage contributes to cancer progression and metastasis in multiple ways. Thus, the disruption of integrin/Src/FAK signaling may contribute to increased cancer cell death and metastatic phenotype [41]. Importantly, Src kinases are overexpressed in a variety of human tumors, and increased Src activity often correlates with malignant potential and metastasis of pancreatic cancer and many other tumors, as it plays an integral part of cellular migration, proliferation, adhesion, and angiogenesis. Therefore, Src kinases have been proposed as a molecular target for therapy in such cancers [42], [43]. Src is activated by the phosphorylation of Tyr-416 and dephosphorylation at the negative regulatory site Tyr-527 [44]. Activation of FAK, by integrin clustering, leads to auto-phosphorylation at Tyr-397, the binding site for the Src family kinases [24]. Therefore, we investigated the activity of Src (p-Tyr-416) and FAK (p-Tyr-397) after targeting 5-HT_1B_ and 5-HT_1D_ receptors. In correlation with β1 integrin reduction, silencing the expression of 5-HT_1B_ and 5-HT_1D_ receptors, lead to profound reductions of active Src and active FAK in both PANC-1 cells (Fig. 4A) and MIAPaCa-2 cells (Fig. 4B). This data suggests that 5-HT_1B_– and 5-HT_1D_–mediated signaling is involved in activation β1 integrin/Fak/Src complex. Inhibition of this engagement cascade may be one of the underlying mechanisms that mediate inhibition of PaCa cell invasion and proliferation evolved after targeting 5-HT_1B_ and 5-HT_1D_ receptors.

### 5-HT_1B_ and 5-HT_1D_ receptors regulate ECM/uPAR/MMP-2 signaling

A prerequisite of the ability of a cancer cell to undergo metastasis into distant tissues is to penetrate surrounding extracellular matrices (ECM) [27]. Plasma membrane urokinase-type plasminogen activator receptor (uPAR) is a protein that binds with high-affinity and activates the serine protease uPA, thus regulating proteolytic activity at the cell surface [45]. Plasmin activated by uPA can break down ECM directly or degrade the ECM indirectly through activation of pro-matrix metalloproteinases (MMPs) [46]. Although plasmin has been shown to principally activate MMP-1, -3, and -9, increasing evidence proves that uPA/plasmin can activate pro-MMP-2 and thereby promoting tumor invasion and metastasis [46], [47]. Thus, we examined whether 5-HT_1B_ and 5-HT_1D_ receptors regulate the expression of uPAR/MMP-2, as important markers for cell invasion [45]. Knockdown of such receptors results in significant reductions in the expression level of both ECM proteins in both PANC-1 and MIAPaCa-2 cells (Fig. 4A and B respectively).

### 5-HT_1B_ and 5-HT_1D_ receptors regulate the zinc finger transcriptional factors of epithelial mesenchymal transition (EMT)

Epithelial-to-mesenchymal transition (EMT) is implicated in the progression of primary tumors towards metastasis, and is likely caused by a pathological activation of transcription factors regulating EMT [48]. This critical process requires a loss of cell-cell adhesion, as well as the acquisition of a fibroblastoid motile phenotype [49]. To explore the role of 5-HT_1B_ and 5-HT_1D_ receptors in regulating EMT-related regulators, we investigated the expression of the zinc finger ZEB1 transcriptional factors that repress the cell–cell junctions [50], after knock-down of these receptors, using Western blot analysis. We investigated the expression of TCF8, as one of the EMT-inducing transcription factors belongs to ZEB1 family, which triggers epithelial dedifferentiation by impairing the expression of E-cadherin, the epithelial adhesion protein and the key mediator of cell–cell junctions [48]. The EMT tight junction proteins, claudins, were identified as potent inhibitors of the invasiveness and metastatic phenotype of pancreatic cancer cells [51]. These tight junctions are the most apical components of intercellular junctional complexes in epithelial and endothelial cells. They separate the apical and baso-lateral cell surface domains, maintaining cell polarity [38]. The loss of tight junction function leads, in a part, to invasion of cancer cells [52], and enhances tumor cell proliferation [48]. In particular, claudin-1 plays crucial roles in epithelial cell polarity during EMT [38]. Thus, we also examined the levels of Snail, the important transcriptional repressor of both E-cadherin [50], and claudin-1 [38], [53]. Our results demonstrated that down-regulation of 5-HT_1B_ and 5-HT_1D_ receptors significantly reduce the expression of TCF8/ZEB1 and Snail, indicating the effective inhibition of EMT. The basal level of E-cadherin expression in untreated cells was un-detectable by Western blotting, probably due to the high basal expression of TCF8/ZEB1, which impairs the E-cadherin expression. Because Snail acts as transcription repressor of the genes of claudin/occludin, EMT tight junction proteins [53], we further investigated the expression of claudin-1, which is known to be expressed in various types of epithelial cells, playing an important role in epithelial cell polarity, cancer invasion and metastasis [54], [55]. Parallel to the reduction of the Snail and TCF8/ZEB1 levels, down-regulation of 5-HT_1B_ and 5-HT_1D_ receptors was accompanied with obvious up-regulation of claudin-1 expression in both PANC-1 and MIAPaCa-2 cells (Fig. 4C and D). Furthermore, as shown by our RPPA data (Fig. 4E), the inhibition of the expression of the 5-HT_1B_ and 5-HT_1D_ receptors was associated with up-regulation of E-cadherin, as well as decrease in its transcriptional repressor, TWIST, one of the transcription factors that modulate EMT, along with Snail1, Slug, ZEB1, ZEB2, E12, E47 [50]. In conclusion, targeting 5-HT_1B_ and 5-HT_1D_ receptors mediate inhibition of EMT attainment and induce epithelial molecular characteristics.

### TG2/NF-κB signaling underlies 5-HT_1B/1D_ receptors-mediated pathways promoting proliferation and invasion

**Figure 5:**
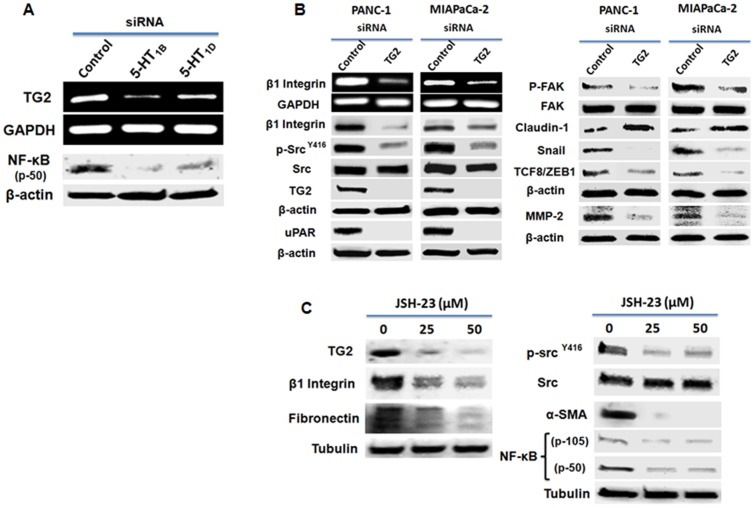
5-HT_1B/1D_ receptors-induced regulation of TG2/NF-κB signaling might mediate proliferation/invasion-promoting pathways. (**A**) 5-HT_1B_ and 5-HT_1D_ receptors regulate TG2 and NF-κB expression. PANC-1 cells were treated with indicated siRNAs as described above. (**B**) siRNA-mediated TG2 knockdown induced downstream molecular effects similar to that observed after knockdown of 5-HT_1B_ and 5-HT_1D_ receptors expression. Cells were transfected with 50 nM of TG2 siRNA or control siRNA, and cell lysates were subjected to Western blot analysis. β-actin was used as loading control. (**C**) Inhibition of constitutive activation of NF-κB inhibits the key signaling promoting proliferation/invasion and decreases the mesenchymal markers, Fibronectin and α-SMA. Cells were treated with indicated concentration of NF-κB activation Inhibitor II, JSH-23, for 24 h, and cell lysates were subjected to Western blot analysis. Tubulin was used as loading control. Three independent experiments were performed with similar results, and representative data is shown.

Tissue transglutaminase (TG2) is implicated in regulation of cell attachment, interactions of the cells with the surrounding ECM, motility, invasion and is considered as a bad prognostic factor in different cancers, including PaCa [50], [56], [57], [58]. Importantly, TG2 can exist in complex with integrins (β1, β4 and β5) in cancer cell membranes [26]. It also stabilizes the β1-integrin/Fibronectin complex at the cell surface to support cell adhesion, motility and invasion [57]. Beside modifying ECM proteins interactions, extracellular TG2 was found to activate nuclear factor-kappa B (NF-κB) signaling, leading to CD44 up-regulation and EMT activation, contributing to increased cancer cell invasiveness and peritoneal dissemination [59]. On the other hand, intracellular TG2 is known to modulate intracellular signaling including FAK, Akt [60], and NF-κB signaling [61], to promote proliferation and cell adhesion. Interestingly, constitutive NF-κB activation is known to play a key role in the aggressive behavior of pancreatic cancer [62]. The tight correlation between TG2 expression/activation and constitutive activation of NF-κB in PaCa cells and many other cancers [61], warranted our attention to address the question whether 5-HT_1B/1D_ receptors-activated proliferation/invasion signaling pathways is mediated through TG2 and/or NF-κB. Thus, we first examine if 5-HT_1B_ and 5-HT_1D_ receptors regulate the TG2/NF-κB expression. Our results show that down-regulation of 5-HT_1B_ and 5-HT_1D_ receptors significantly reduce the expression of both TG2 and NF-κB (Fig 5A), suggesting that 5-HT_1B/1D_ receptors lie as an upstream of TG2/NF-κB complex. We next knocked TG2 down and examined the same molecular targets that are regulated by 5-HT_1B/1D_ receptors and involved in proliferation, invasion and EMT process. Consistent with the events that produced after down-regulation of HT_1B/1D_ receptors, knockdown of TG2 induces similar trend (Fig. 4B), further supporting the role of TG2 in regulation proliferation and invasion pathways in PaCa cells. In fact, there is a direct correlation between TG2 expression and NF-κB activation in various cancer cell lines [61], and NF-κB activation is a known inducer of EMT in TG2-expressing cells [50], [63]. TG2 and NF-κB form a recruitment complex at to the promoter sequence of Snail leading to its transcriptional regulation, facilitating EMT attainment [50]. Because of this positive cross-linking and regulatory loop, we next examined the effect of specific inhibition of constitutive NF-κB activation in PaCa cells, using a specific NF-κB activation inhibitor, JSH-23. Treating the cells with JSH-23 led to a concentration-dependent decrease in TG2 expression, suppression of β1 integrin/Src signaling along with inhibition of EMT markers α-SMA and Fibronectin. Parallel to our results, NF-κB was recently found to regulate β1 integrin expression in breast cancer cells [64]. Taken together, this data suggest a close link between TG2/NF-κB, and 5-HT_1B/1D_ receptors in mediating pro-tumorigenic pathways and in contributing to PaCa cells proliferation and invasion. It also suggest a rationale for inhibiting endogenous 5-HT_1B/1D_ receptors to inhibit TG2/NF-κB axis, leading to suppression of the potential cellular tumorigenic downstream signaling.

**Figure 6:**
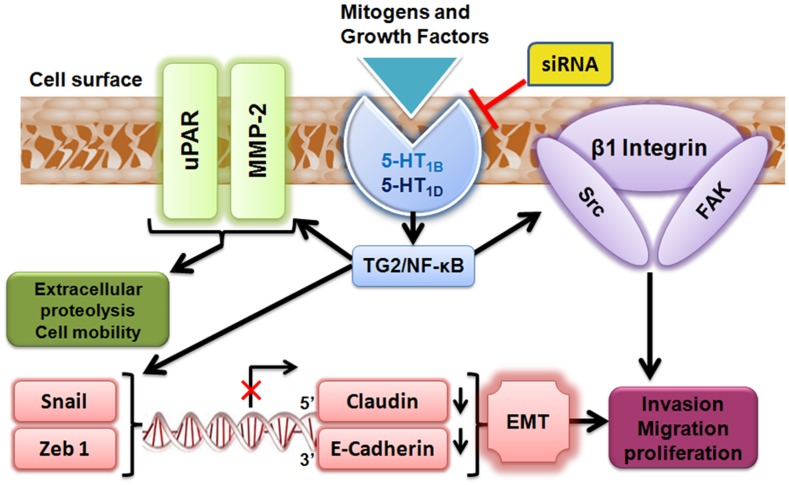
The postulated molecular regulation of 5-HT_1B_ and 5-HT_1D_ receptors to β1 integrin/Src/FAK complex, ECM/uPAR/MMP-2 signaling and the zinc finger transcriptional regulators of EMT in PaCa cells. These receptors-regulated signaling might be mediated through TG2/NF-κB axis. 5-HT_1B_ and 5-HT_1D_ receptors mediate β1-integrin activity to recruit a Src–FAK complex promoting the cell proliferation and migration. Upon different extracellular mitogenic stimuli (e.g; growth factors, hormones and neurotransmitters), the over-expressed 5-HT_1B/1D_ receptors promote the activation of urokinase plasminogen activator receptor (uPAR), and matrix metalloproteinase (MMP-2), facilitating extra-cellular matrix degradation and enhancement of invasion process. Also, 5-HT_1B/1D_ receptors stimulate the expression of zinc finger transcriptional factors (Snail and TCF8/ZEB1). These mesenchyme markers, in turn, repress the gene transcription of epithelial markers (claudin-1 and E-cadherin) leading to stimulation of epithelial mesenchymal transition (EMT), further supporting the proliferation and invasiveness of PaCa cells.

Figure 6 depicts a summary of the suggested molecular mechanisms of the down-regulation of 5-HT_1B_– and 5-HT_1D_–mediated inhibition of proliferation and invasion of PaCa cells.

## Discussion

The essential phenotype of epithelial cancers (e.g., pancreas, breast, prostate) includes: self-sufficiency for growth signals, sustained tissue invasion and insensitivity to growth inhibitors [16], [65]. Pancreatic cancer is one of the most deadly neoplastic diseases because it typically diagnosed at an advanced stage. At the time of diagnosis, most pancreatic cancers are, therefore, inoperable and have metastasized to distant organs. In addition, this malignancy is generally unresponsive to conventional radio- and chemotherapy, resulting in a mortality rate near 100% within 6 months of diagnosis [4]. In the current study, we demonstrated the over-expression of 5-HT_1B_ and 5-HT_1D_ receptors subtypes in PaCa cells, determined their involvement in PaCa cells progression, and investigated the effect of down-regulation of these receptors on PaCa cells growth and invasion. We showed that 5-HT_1B_ and 5-HT_1D_ receptors promote the key survival factors involved in supporting PaCa cells proliferation and invasion and thus they may be considered as one of the novel potential therapeutic targets in PaCa treatment.

The biogenic amines (e.g; serotonin, 5-HT) have been reported to be released as growth factors in different malignancies [16]. 5-HT can exert its mitogenic effect in interaction with other hormones or growth factors [22], [66]. It has been previously shown that different 5-HT receptors can trigger the proliferative properties of 5-HT and mediate cascades of mitogenic signaling pathways [67]. The stimulation of 5-HT receptors leads to transcription factors phosphorylation and cell division activation, which is a common reaction to many extracellular stimuli, including growth factors, hormones and neurotransmitters in mammalian cells, such as human pancreatic carcinoid, and mouse fibroblast cells [68], [69]. In prostate cancer, these growth factors act on the adjacent (exocrine) tumor cells to enhance tumor growth, differentiation and angiogenesis, the events that lead to a poor prognosis [18], [70]. Recently, 5-HT_1B_ receptors were associated with an increased proliferation index, correlated with the size of the tumor in hepatocellular cancer patients [71], and were shown to play a potential role in colorectal cancer [69]. Since we showed here that the 5-HT_1B_ and 5-HT_1D_ receptors are highly over-expressed in PaCa cells, we tried to explore the etiological associations between its expression and PaCa progression.

Mechanisms responsible for elaboration of growth- and invasion-stimulating signals and molecular events responsible for their sensing are deregulated in cancer cells. To get insight into the role of these 5-HT-1 receptors in PaCa progression, we investigated some proliferation and invasion biomarkers, in order to speculate some of the contributing transduction signaling mediated by these receptors in PaCa cells. uPAR was proposed as an important regulator of the invasive properties of cancer cells. It is highly expressed in virtually all human cancers, and such over-expression is mediated by some extracellular matrix proteins [45], [72]. The over-expression of the uPA/uPAR-system components correlates with increased proliferation, migration, and invasion affecting the malignant phenotype of the cancer [45]. Invasive tumor cells have a marked ability to degrade extracellular matrix via activation of matrix metalloproteases (MMPs). MMP-2 rather than MMP-9 was activated in the metastatic pancreatic cancer, and it is secreted as an inactive zymogen and requires distinct activation processes [46]. MMP-2 can be activated through plasminogen activator/plasmin system, in which pro-uPA binds to its receptor, uPAR [73], resulting in uPA activation, acceleration of the conversion of plasminogen to plasmin on the cell surface, and localization these enzymes to focal contact sites [46], [74]. Our data shows the effective down-regulation of uPAR and MMP-2 following the silencing of 5-HT_1B_ and 5-HT_1D_ receptors, supporting the notion of the distinct correlation between the expression of these receptors and the expression of uPAR/MMP-2. Although the involvement of uPA cascade in MMP-2 activation was previously reported in metastatic pancreatic cancer BxPc3 cells [46], we showed for the first time that the expression pattern of these proteins is regulated by 5-HT_1B/1D_ receptors, in the metastatic PANC-1 and MIAPaCa-2 cells.

On the other hand, the uPAR-mediated activity requires integrin-dependent signaling [75], [76]. The integrin family of cell adhesion molecules facilitates the penetration and invasion of the cancer cell to the surrounding extracellular matrices [27]. In pancreatic BxPC3 cells, the blocking of the over-expressed β1 integrin was found to decrease the activation of uPA/MMP-2 with subsequent decrease in metastasis [46]. β1 integrin is proposed as an emerging target that limits the metastasis of the tumors *in-vivo* [77], since it plays a profound role in cancer initiation, tumor growth progression and invasion/metastasis, through cell binding to ECM [78], [79]. Moreover, β1 integrin expression is known to induce Src activity, which is associated with shorter patient survivals, making both β1 integrin and Src appealing targets for cancer therapy [80]. Src is one of the tyrosine kinases that plays a critical role in signal transduction associated with cell–extracellular matrix interactions, migration and adhesion [81]. Interestingly, Src inhibitors have shown a significant inhibition of the tumor growth in a subset of human pancreatic tumor xenografts [82]. In addition, recruitment of Focal adhesion kinase (FAK)/Src complex mediates and regulates the signaling events downstream of integrin-dependent pathway [27]. The cytoplasmic protein tyrosine kinase, FAK, is involved in integrin-mediated signal transduction and plays an important role in the control of cell spreading, migration, and survival [83]. Src/FAK mutually regulates the activity of each other and promotes normal and cancer cell migration by regulating focal adhesion formation and turnover through multiple signaling connections [41]. Enhanced FAK signaling was also connected to elevated uPA expression, directly contributing to the proliferation, invasion and metastatic phenotype [84]. It was documented that the cells expressing an activated FAK–Src signaling alter the surrounding stromal environment, facilitating breakdown of cell-cell adhesion, increased cell-matrix– and focal–adhesions and tissue invasion. Accordingly, inhibition of Src/FAK activity leads to restoration of cell-cell adhesion and inhibits cell migration and invasion [41]. In the current study, we demonstrated the potential inhibition of β1 integrin protein and gene expression and the inhibition of Src/FAK activity after silencing of 5-HT_1B_ and 5-HT_1D_ receptors in PANC-1 and MIAPaCa-2 cells (Fig. 4 A-B). Furthermore, Integrins serve as receptors for some ECM proteins (e.g., Fibronectin, Vitronectin, Laminin and Collagen) [26]. The cell attachment/motility regulatory protein, tissue transglutaminase (TG2), was found to play a role in stabilizing the β1-integrin/Fibronectin complex [84], [85]. We showed here that molecular downstream signaling mediated through TG2 is similar to that regulated by 5-HT_1B_ and 5-HT_1D_ receptors, and that the inhibition of 5-HT_1B_ and 5-HT_1D_ receptors suppressed the expression of TG2 and NF-κB (Fig. 5A and B). In many types of tumor cells including PaCa, TG2 contributes to constitutive activation of NF-κB, which, in turn, activates gene transcription [61]. Intracellular TG2 promotes degradation of IκBα resulting in constitutive activation of NF-κB through a canonical pathway, via the formation of the p52/RelB complex and its translocation to the nucleus [59]. Alternatively, association of TG2 with p65/p50 complex could mitigate the binding of IκBα to NF-κB complex, resulting in NF-κB constitutive activation. TG2-mediated NF-κB activation/nuclear translocation can result in constitutive activation of transcription of various target genes, including TG2 [61]. In addition, NF-κB was recently found to bind at the β1 integrin promoter region in breast cancer cells, leading to significant increase in β1 integrin expression [64]. This indicates a regulatory role for NF-κB to β1 integrin and subsequent downstream signaling (eg., Src, FAK, etc) as well as other recruited proteins involved in cell attachment and motility. Parallel with these results, our current data demonstrates that specific inhibition of NF-κB led to significant inhibition of β1 integrin/Src activation signaling and other β1-integrin-associated ECM proteins (e.g. TG2 and Fibronectin) (Fig. 5C).

Tumor invasion appears to be also controlled by other coordinated series of complex cellular and molecular processes that enable tumor cells to dissociate and migrate from the primary tumor [86]. The changes in cell adhesion and migration during tumor invasion are reminiscent of EMT; a process that reorganizes epithelial cells to become migratory mesenchymal cells. EMT is a crucial process in tumor progression providing tumor cells with the ability to escape from the primary tumor, to migrate to distant regions and to invade tissues, promoting oncogenic progression and metastasis [28]. Alterations of EMT transcription factors define consequences for tumorigenesis [87]. The zinc finger ZEB1 EMT-inducing factor, TCF8, has been identified as a potent transcriptional repressor of E-cadherin, the protein that rescues epithelial architecture [48]. Snail is another central EMT inducer, by its inhibitory effect on both E-cadherin and other EMT-tight junction proteins [50], [53]. Snail directly binds to the promoters of claudin/occludin genes, resulting in repression of the promoter activities and loss of epithelial cell polarity [53]. These EMT-molecular triggers repress genes encoding cadherins, claudins, cytokines, integrins, mucins and occludin proteins, thereby promoting EMT [56]. In addition, Snail confers resistance to cell death [88]. In pancreatic cancer, expression of claudin-4 was associated with significant reduction of *in-vitro* invasive potential, inhibition of colony formation, as well as reduction of *in-vivo* metastases [51]. Thus, it is thought that the loss of tight junction function leads, in a part, to invasion and metastasis of cancer cells [52]. The functional loss of epithelial cell polarity also enhances tumor cell proliferation [48]. Here, we show that targeting 5-HT_1B_ and 5-HT_1D_ receptors was associated with up-regulation of E-cadherin (the fundamental key adhesion and epithelial marker), and claudin-1 expression, along with concomitant decreases in the expression levels of the E-cadherin–repressors, TCF8/ZEB1, Snail and TWIST (Fig. 4C-E).

In conclusion, the *in-vitro* data pioneered by our study suggests the involvement of 5-HT_1B_ and 5-HT_1D_ receptors in the activation of the β1 integrin-mediated proliferation/invasion promoting signaling and EMT process, representing these receptors as novel important regulators of pro-tumorigenic signals. To have generally accepted view, we are currently investigating the value of *in-vivo* targeting 5-HT_1B_ and 5-HT_1D_ receptors in nude mice-bearing PANC-1 tumor xenografts using siRNA-nanoparticles, to demonstrate the role of these receptors in pancreatic tumor progression, in order to establish a novel and efficient molecularly targeted therapy for pancreatic cancer.

## Supporting Information

Figure S1 **Effects of dual down-regulation of 5-HT_1B_ and 5-HT_1D_ receptors on PaCa cell proliferation.** PANC-1 cells were transfected with control, 5-HT_1B_ or 5-HT_1D_ siRNAs, or transfected with both 5-HT_1B_ and 5-HT_1D_ simultaneously. After 72 h, proliferation was evaluated by an MTS assay. Data are represented as mean ± SD of three independent experiments. * P<0.05 vs. control cells. # represents significant difference between indicated groups (P<0.05).
(TIF)
