# Supplementary figures and images for: Down-Regulation of 5-HT1B and 5-HT1D Receptors Inhibits Proliferation, Clonogenicity and Invasion of Human Pancreatic Cancer Cells
Source: PLoS One. 2014 Aug 29;9(8):e105245. doi: 10.1371/journal.pone.0105245 (PMC4149367; doi:10.1371/journal.pone.0105245)

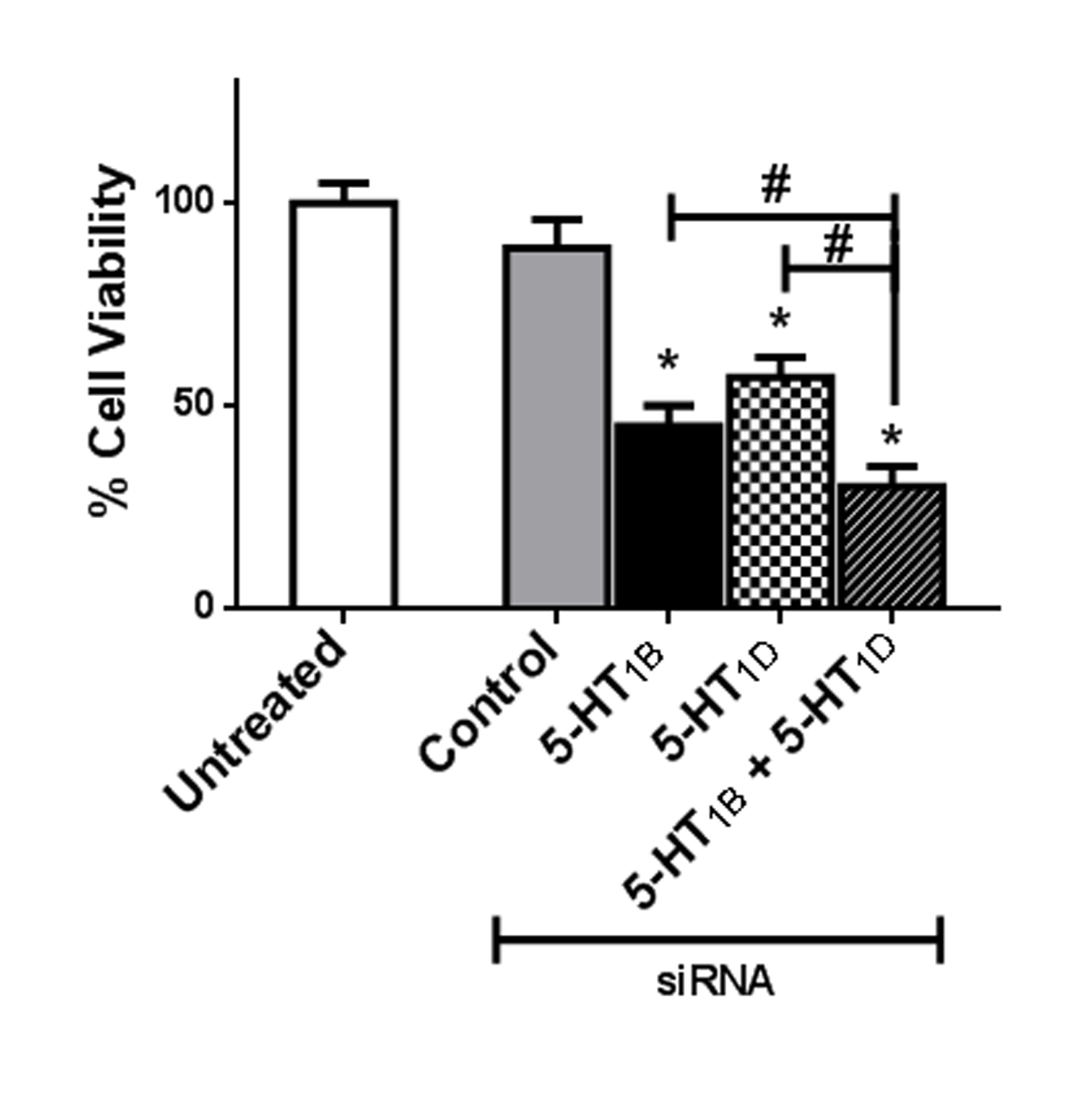

Supplement: Figure S1 — Effects of dual down-regulation of 5-HT1B and 5-HT1D receptors on PaCa cell proliferation. PANC-1 cells were transfected with control, 5-HT1B or 5-HT1D siRNAs, or transfected with both 5-HT1B and 5-HT1D simultaneously. After 72 h, proliferation was evaluated by an MTS assay. Data are represented as mean ± SD of three independent experiments. * P<0.05 vs. control cells. # represents significant difference between indicated groups (P<0.05). (TIF) [file pone.0105245.s001.tif]
